# First report on treating spontaneous infectious spondylodiscitis of lumbar spine with posterior debridement, posterior instrumentation and an injectable calcium sulfate/hydroxyapatite composite eluting gentamicin: a case report

**DOI:** 10.1186/s13256-016-1125-y

**Published:** 2016-12-12

**Authors:** Richard Bostelmann, Hans Jakob Steiger, Armin O. Scholz

**Affiliations:** 1 Department of Neurosurgery, University Hospital of Düsseldorf, Düsseldorf, 40225 Germany; 2 Department of Trauma and Hand Surgery, University Hospital of Düsseldorf, Düsseldorf, 40225 Germany

**Keywords:** Spondylodiscitis, Surgical treatment, Posterior instrumentation, Local antibiotic, Injectable calcium sulfate/hydroxyapatite, Gentamicin, Vertebral osteomyelitis, Case report

## Abstract

**Background:**

Spontaneous infectious spondylodiscitis is a rare, but serious disease with the risk of progressive neurological impairment. The surgical approach to spontaneous infectious spondylodiscitis is in most cases an anterior debridement and fusion, often in staged surgeries. Here we report a case of single-stage posterior debridement and posterior instrumented fusion in combination with an injectable calcium sulfate/hydroxyapatite composite eluting gentamicin.

**Case presentation:**

A 59-year-old Caucasian man presented with a 6-week history of lumbar pain without sensory or motor disorders of his lower extremities. A magnetic resonance imaging scan of his lumbar spine in T2-weighted sequences showed a high signal of the intervertebral disc L4/L5 and in T1-weighted sequences an epidural abscess at the posterior wall of L4. Additional computed tomography imaging revealed osteolytic destruction of the base plate of L4 and the upper plate of L5. Antibiotic therapy was started with intravenous ciprofloxacin and clindamycin. We performed a posterior debridement via a minimally invasive approach, a posterior percutaneous stabilization using transpedicular screw-rod instrumentation and filled the intervertebral space with an injectable calcium sulfate/hydroxyapatite composite which elutes a high concentration of gentamicin. The patient’s lower back pain improved quickly after surgery and no recurrence of infection has been noticed during the 1-year follow-up. Computed tomography at 11 months shows complete bony fusion of L4 and L5.

**Conclusions:**

An injectable calcium sulfate/hydroxyapatite composite releasing a high level of gentamicin can support the surgical treatment of spondylodiscitis in combination with posterior debridement and transpedicular screw-rod instrumentation.

## Background

Infectious spondylodiscitis is usually secondary to spinal surgery. Spontaneous infectious spondylodiscitis (SIS), caused by the hematogenous spread of bacteria, is a relatively rare disease. However, a rise in the incidence of SIS has recently been noticed due to increasing life expectancy, use of endovascular devices, diabetes mellitus, and HIV [1, 2].

Conservative treatment of SIS is effective in most patients [3], but surgical treatment is advocated in cases of poor response to conservative treatment, progressive neurologic impairment, spinal instability, or progressive bone alteration [4–6]. Anterior debridement and fusion [7], mostly in a staged approach [8] are usually suggested. Here we report a case in which an injectable, antibiotic-eluting bone graft substitute (BGS) was used to facilitate fusion in single-stage posterior debridement and posterior instrumentation.

## Case presentation

**Fig. 1:**
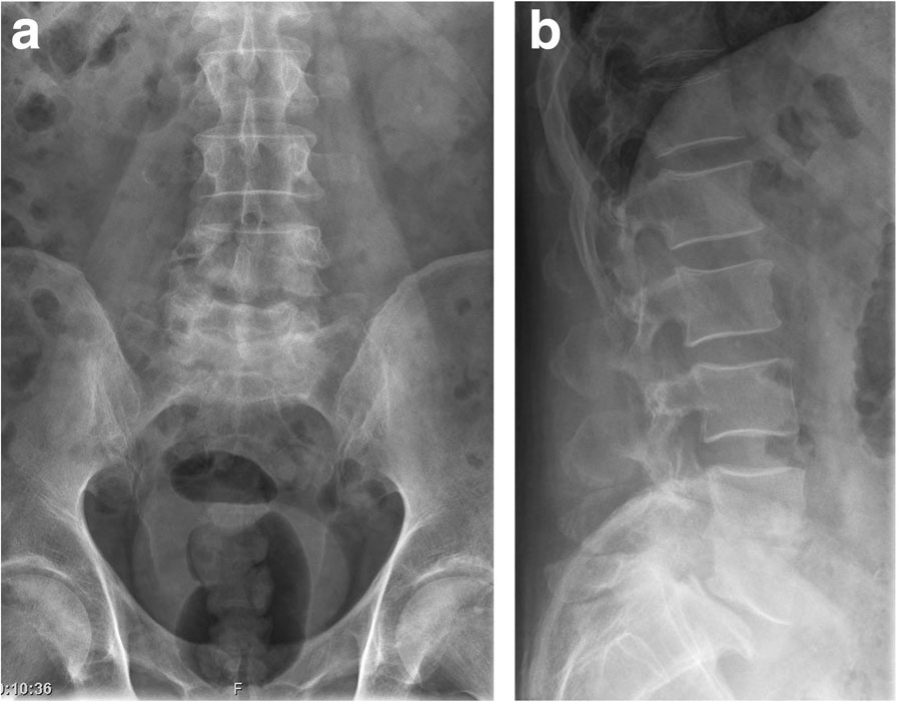
**a** and **b** Preoperative radiograph of the lumbar spine anterior and lateral on 6 Jan 2016: narrowing of the intervertebral space L4 and L5 with osseous destruction of base plate L4 and upper plate L5

**Fig. 2:**
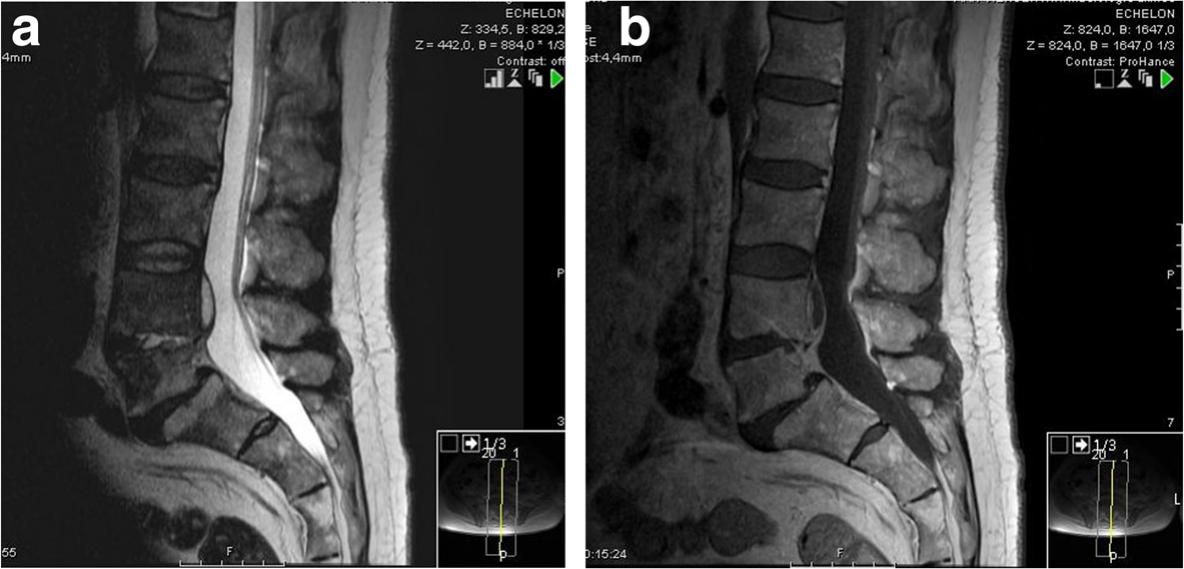
**a** and **b** Preoperative magnetic resonance imaging of the lumbar spine on 30 Dec 2014 T1- (*right*) and T2- (*left*) weighted sequences: note the enhanced signal of the intervertebral disk L4/L5 and the epidural abscess at the posterior wall of L4

**Fig. 3:**
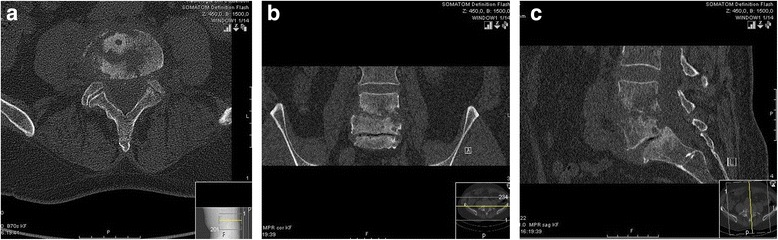
**a**, **b** and **c** Preoperative computed tomography scan of the lumbar spine on 6 Jan 2015 (axial (*left*), coronary (*middle*) and sagittal (*right*) reconstructions): significant bone osteolysis and erosion of base plate L4 and upper plate L5

A 59-year-old Caucasian man presented at our university hospital with a 6-week history of lumbar pain without sensory or motor disorders of his lower extremities. The pain had not responded to the common conservative treatment of lower back pain [nonsteroidal anti-inflammatory drugs (NSAIDs), physiotherapy, etc.] [9]. Our patient had a history of diabetes mellitus (noninsulin-dependent) but was otherwise healthy. A physical examination showed pressure pain and tapping tenderness at the lower lumbar spine. In blood biochemistry, an elevated C-reactive protein (CRP: 27 mg/L) and a normal white blood cell count (WBC: 6.8 * 10^9^/L) were found. Plain radiographs of the lower spine revealed a narrowing of the intervertebral space between L4 and L5 with irregularity of the endplates (Fig. 1). Magnetic resonance imaging (MRI) of the lumbar spine in T2-weighted sequences showed a high signal of the intervertebral disc L4/L5 and in T1-weighted sequences an epidural abscess at the posterior wall of L4 (Fig. 2). Additional computed tomography (CT) imaging revealed osteolytic destruction of the base plate L4 and the upper plate L5 (Fig. 3). Antibiotic therapy had been started with intravenous ciprofloxacin and clindamycin. Because of the progressive bone destruction of the base plate L4 and upper plate L5, we performed a posterior debridement via a minimally invasive dorsolateral approach, a posterior percutaneous stabilization using transpedicular screw-rod instrumentation and filled the intervertebral space with an injectable BGS, which elutes a high concentration of gentamicin (CERAMENT™ G, Bonesupport, Lund, Sweden) after removing the pathological disc tissue. For posterior monosegmental instrumentation the Viper® 2 system (DePuy Synthes, Umkirch, Germany) with four polyaxial screws (6 × 45 mm each) and two rods (40 mm each) were used. The epidural abscess was not evacuated, since it did not compress the cauda equine.

**Fig. 4:**
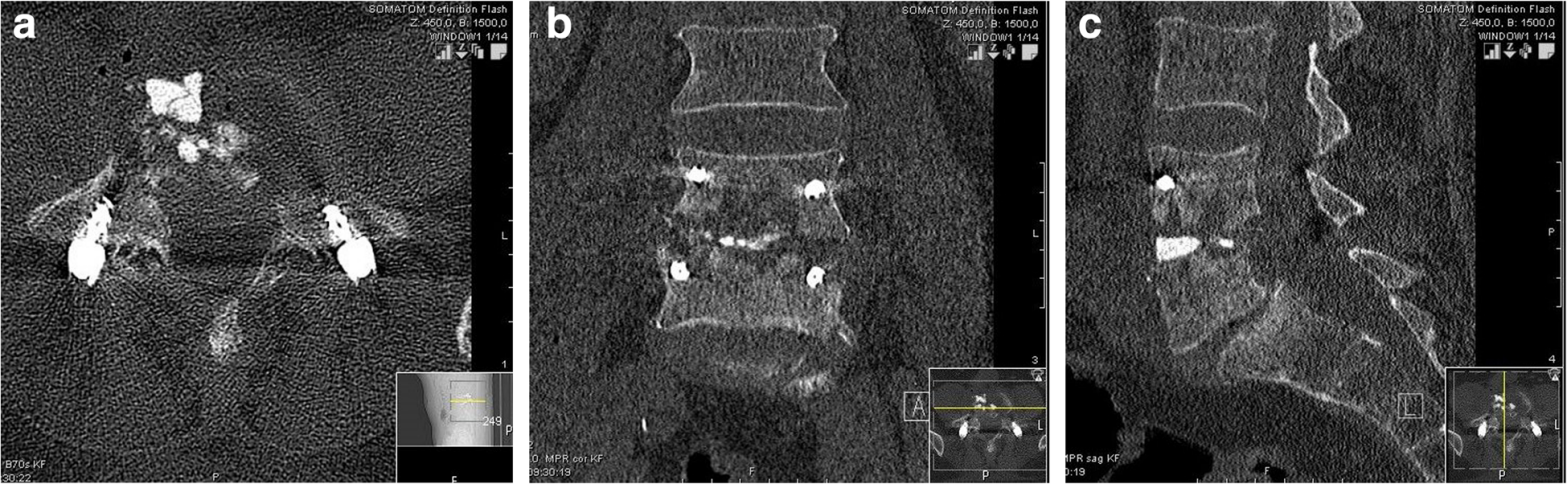
**a**, **b** and **c**: Postoperative computed tomography scan on 9 Jan 2015 (axial (*left*), coronary (*middle*) and sagittal (*right*) reconstructions): correct position of transpedicular screw-rod instrumentation. Intervertebral space was filled with the antibiotic-eluting bone graft substitute, which contains a radio contrast agent (Iohexol) with good visibility (especially on sagittal reconstruction)

A biopsy of the intervertebral disk was sent for microbiological evaluation, but a causative bacteria could not be detected. Our patient’s lower back pain improved quickly after surgery. A postoperative CT scan on day 3 confirmed the correct positioning of the transpedicular screw-rod instrumentation (Fig. 4). The antibiotic-eluting BGS is clearly visible in the intervertebral space (especially on the sagittal reconstruction, Fig. 4). Ciprofloxacin and clindamycin were continued for 4 weeks intravenously, followed by 4 weeks of oral administration. The surgical incision healed *ad primam intentionem* without prolonged wound drainage. At discharge from hospital 4 weeks after surgery our patient was ambulatory, and CRP (0.8 mg/L) and WBC (8.1 * 10^9^/L) levels were in the normal range.

**Fig. 5:**
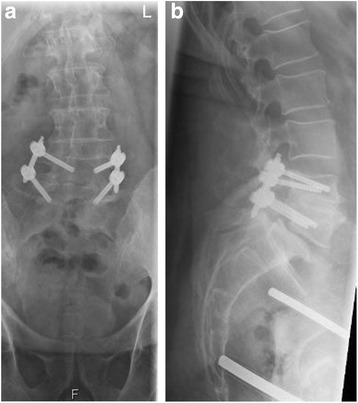
**a** and **b** Follow-up radiograph of the lumbar spine anterior-posterior and lateral at 11 months on 7 Dec 2015: unchanged position of the posterior instrumentation. Bony fusion of L4 and L5

**Fig. 6:**
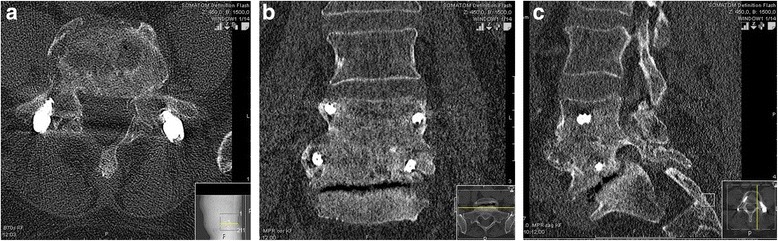
**a**, **b** and **c** Follow-up computed tomography scan of the lumbar spine at 11 months on 7 Dec 2015: consistent position of the posterior instrumentation. Complete bony fusion of L4 and L5

No recurrence of infection was noticed during the 1-year follow-up. Our patient was generally well without restrictions in his daily activities and able to work in his previous job. He complained about mild pain from the left lower spine to the left dorsal leg from time to time. He was able to walk without pain for 45 minutes. Radiography and CT of his lower spine during follow-up examination at 11 months show complete bony fusion of L4 and L5 (Figs. 5 and 6).

## Discussion

To the best of our knowledge, this is the first case where an injectable, gentamicin-eluting BGS was used to facilitate fusion in the treatment of SIS. The presentation of our patient was quite typical for a SIS: mean age about 50 years [10], back pain not responding to nonsurgical treatment [11], and elevation of inflammatory markers (CRP) [10]. The preferred diagnostic tool is an MRI scan, with 76% definite and 20% possible diagnosis of SIS, if the patient presents with symptoms that have lasted longer than 2 weeks [12]. Operative treatment of SIS is indicated in cases of poor response to conservative treatment, progressive neurologic impairment, spinal instability, or progressive bone alteration [4–6]. There is still an ongoing debate about the most suitable surgical approach. Some spine surgeons prefer an anterior approach with debridement, fusion with autograft, and anterior or posterior instrumentation [13, 14]. However, a minimally invasive posterior approach might be less exhausting for the patient. Moreover, debridement of the posterior intervertebral space and the epidural abscess might be easier via the posterior approach [15]. Independent of the surgical approach, usually autogenous bone grafts are considered to be the “gold standard” in spine reconstruction [7, 16]. However, some well-recognized complications associated with graft harvesting from the iliac crest including pain at the donor site, nerve injury, hematoma, infection, and pelvis fracture have to be taken into account [16–19]. These risks could be avoided with the use of a synthetic BGS. Usually, the use of a synthetic BGS is not indicated in septic or post-septic sites due to the risk of a foreign body contamination as a trigger of recurring infection. Therefore, the combination of a calcium sulfate/hydroxyapatite composite with local antibiotic gentamicin (CERAMENT™ G, Bonesupport, Lund, Sweden) seemed to be a reasonable alternative. So far, the applied BGS in our case has been used in bone reconstruction after osteomyelitis [20], but not in spine surgery. The composite enabled us to combine posterior debridement, posterior stabilization, and filling of the intervertebral space in a one-stage procedure. Antibiotics were administered for 4 weeks intravenously, followed by a 4-week course of oral administration, as suggested by Zhang *et al*. [10].

The administration of local anti-infective substances is becoming more popular in the treatment of SIS.

Other groups have used antibiotic bone cement beads [21], a combination of antibiotic-impregnated BGS and autograft [22] or bioactive glass S53P4 [23]. In our opinion, the advantage of the injectable calcium sulfate/hydroxyapatite composite plus gentamicin is the high local concentration of gentamicin at the desired location [24], the complete resorption of the BGS, and its osteoconductivity.

## Conclusions

An injectable calcium sulfate/hydroxyapatite composite eluting a high level of gentamicin can support the surgical treatment of spondylodiscitis in combination with posterior debridement, transpedicular screw-rod instrumentation, and systemic antibiotic therapy. A CT scan confirmed complete fusion after 11 months.
